# Cisplatin plus pemetrexed therapy and subsequent immune checkpoint inhibitor administration for malignant peritoneal mesothelioma without pleural lesions: Case report

**DOI:** 10.1097/MD.0000000000019956

**Published:** 2020-05-29

**Authors:** Hiroaki Ikushima, Toshio Sakatani, Sayaka Ohara, Hideyuki Takeshima, Hajime Horiuchi, Teppei Morikawa, Kazuhiro Usui

**Affiliations:** a Division of Respirology; b Department of Diagnostic Pathology, NTT Medical Center Tokyo, Tokyo, Japan.

**Keywords:** case report, hyperprogressive disease, immune checkpoint inhibitor, nivolumab, peritoneal mesothelioma

## Abstract

**Rationale:**

Malignant peritoneal mesothelioma is a rare tumor with a poor prognosis and has no recommended therapy after first-line pemetrexed and platinum-based chemotherapy. Moreover, effects of immune checkpoint inhibitors on peritoneal mesothelioma remains to be elucidated. We herein report the case of a 75-year-old man with peritoneal mesothelioma treated with cisplatin plus pemetrexed and subsequent nivolumab.

**Patient concerns:**

A 75-year-old man was referred to our hospital due to lower abdominal pain.

**Diagnosis:**

Positron emission tomography-computed tomography (CT) showed the accumulation of fluorodeoxyglucose in an intraperitoneal mass. A histological examination of a laparoscopic biopsy specimen revealed malignant peritoneal mesothelioma.

**Interventions:**

After 4 cycles of cisplatin plus pemetrexed and 13 subsequent cycles of pemetrexed maintenance therapy showed beneficial responses until CT revealed liver metastasis. Nivolumab was then administered as the second-line therapy.

**Outcomes:**

After 3 cycles of biweekly nivolumab administration, he developed severe abdominal distention. CT revealed an intraperitoneal mass growing much more rapidly than ever, indicating hyperprogressive disease after nivolumab treatment. He ultimately died 51 days after the initial nivolumab administration.

**Lessons:**

To our knowledge, this is the first report of hyperprogressive disease in a case of peritoneal mesothelioma after nivolumab treatment. While immune checkpoint inhibitors may be promising therapeutic strategies for treating malignant peritoneal mesothelioma, careful monitoring must be practiced with their application.

## 1 Introduction

Peritoneal mesothelioma is a rare malignant neoplasm with a poor prognosis.^[1,2]^ Systemic chemotherapy is an important therapeutic strategy for patients with inoperable peritoneal mesothelioma. Cisplatin plus pemetrexed therapy has been reported to be effective for inoperable peritoneal mesothelioma.^[3,4]^ While alternative options or second-line treatments have not been established for peritoneal mesothelioma, the efficiency of the immune checkpoint inhibitor nivolumab as second-line therapy for malignant pleural mesothelioma has been demonstrated in recent studies.^[5,6]^

We herein report the case of a 75-year-old man with peritoneal mesothelioma treated with cisplatin plus pemetrexed and subsequent nivolumab. We obtained informed consent from the patient for reporting this case.

## 2 Case report

A 75-year-old male ex-smoker with a history of asbestos exposure presented with lower abdominal pain. His vital signs were unremarkable, and a physical examination revealed a soft distended abdomen with diminished bowel sounds and slight rebound tenderness in the right lower quadrant. No mass was palpated, and no abnormal findings were found on lung auscultation.

**Figure 1:**
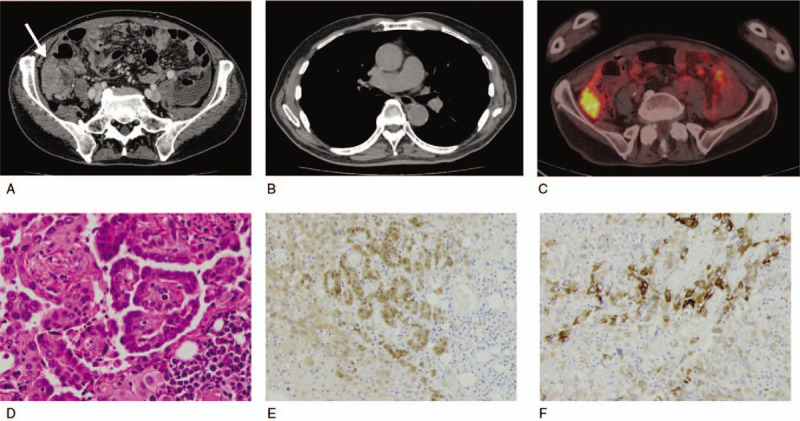
A CT scan on admission showed a large lower abdominal mass (arrow) involving the ascending colon (A). Chest CT (B) and abdominal PET-CT (C) at the time of the diagnosis. Hematoxylin and eosin (D), calretinin (E), and epithelial membrane antigen (EMA, F) staining. CT = computed tomography, PET-CT = positron emission tomography-computed tomography.

Computed tomography (CT) revealed a 50-mm mass involving the ascending colon (Fig. 1A). Multiple peritoneal dissemination nodules and ascites were also observed, while pleural thickening, plaques, or calcification were not detected (Fig. 1B). Positron emission tomography-CT showed the accumulation of fluorodeoxyglucose in an intraperitoneal mass (Fig. 1C). Colonoscopy was performed, but no obvious mass was found on luminal surface of the large intestine. Therefore, to access the mass from the abdominal cavity, a laparoscopic inspection was performed. The abdominal cavity was filled with dark brown ascites, and multiple peritoneal dissemination nodules were located on the greater omentum. A laparoscopic biopsy was performed for the diagnosis of the peritoneal mass and multiple peritoneal dissemination nodules.

Histopathologically, the tumor was entirely composed of round cells with invasion into adipose tissue (Fig. 1D). An immunohistochemical analysis demonstrated that the tumor cells were positive for calretinin, Wilms tumor gene 1, cytokeratin AE1/AE3, epithelial membrane antigen, and Hector Battifora mesothelial epitope-1, while they were negative for carcinoembryonic antigen and Ber-EP4 (Fig. 1E and F). The patient was diagnosed with malignant peritoneal mesothelioma, epithelioid type.

**Figure 2:**
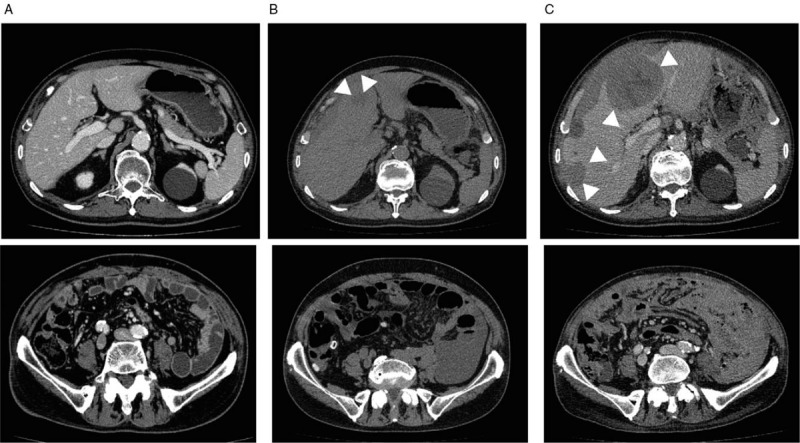
Abdominal CT after 4 cycles of cisplatin plus pemetrexed (A), after 13 cycles of pemetrexed maintenance therapy (B), and after 3 cycles of nivolumab administration (C). Arrowheads indicate liver metastasis. CT = computed tomography.

The patient received 4 cycles of cisplatin 75 mg/m^2^ plus pemetrexed 500 mg/m^2^ as the first-line therapy.^[3,4]^ CT demonstrated a reduction in the right lower abdominal mass, improvement of peritoneal thickening, and clearance of ascites after cisplatin plus pemetrexed treatment (Fig. 2A). Therefore, we started pemetrexed 500 mg/m^2^ maintenance therapy, which was continued without significant adverse effects. However, after 13 cycles of pemetrexed maintenance therapy, CT demonstrated liver metastasis, indicating progressive disease (Fig. 2B). Nivolumab (240 mg/body) was therefore administered as the second-line therapy to emulate pleural mesothelioma treatment.^[5,6]^ After 3 cycles of biweekly nivolumab administration, he developed severe abdominal distention. CT showed that the intraperitoneal lesions and liver metastasis were growing much more rapidly than ever (Fig. 2C), indicating hyperprogressive disease after nivolumab treatment. He ultimately died 51 days after initial nivolumab administration.

## 3 Discussion

We encountered a case of malignant peritoneal mesothelioma without pleural lesions. Mesothelioma is a rare malignant tumor that arises primarily from the mesothelial cells of the serosal membrane lining the pleural, peritoneal, and pericardial cavities. Malignant peritoneal mesothelioma accounts for nearly one-quarter of cases.^[7]^ Patients with peritoneal mesothelioma are more commonly female, younger, and with less frequent asbestos exposure than those with pleural mesothelioma,^[8,9]^ although our present patient showed none of these characteristics. The histopathological subtypes range from epithelioid to sarcomatoid and biphasic types. Among them, epithelioid types have a better prognosis than others.^[10]^

Given the rarity of malignant peritoneal mesothelioma, data on the best treatment strategies have not been fully collected. In the operable stages, cytoreductive surgery and subsequent hyperthermic intraperitoneal chemotherapy have been evaluated in a series of studies and recommended as the standard of care.^[11]^ Systemic chemotherapy is an alternative approach for patients who are inoperable or who wish to pursue non-surgical management. The efficacy of pemetrexed for peritoneal mesothelioma, either alone or in combination with cisplatin, was reported in 2 studies.^[3,4]^ The estimated median overall survival times were 8.7 and 13.1 months for pemetrexed monotherapy and doublet systemic chemotherapy with cisplatin, respectively. Replacing cisplatin with carboplatin demonstrated a similar efficacy, so the carboplatin regimen has been proposed for older patients.^[12]^ Therefore, pemetrexed with cisplatin/carboplatin is considered the standard first-line systemic treatment for peritoneal mesothelioma. However, no therapies have demonstrated a survival benefit as second-line treatment for relapsed or refractory disease.

In the present case, cisplatin plus pemetrexed therapy showed beneficial responses of reducing the right lower abdominal mass, improving the peritoneal thickening, and clearing the ascites. Therefore, we performed pemetrexed maintenance therapy, which also showed a favorable response for about 12 months. In cases of pleural mesothelioma, the immune checkpoint inhibitor nivolumab, an anti-programmed cell death-1 monoclonal antibody, has been shown to be efficacious in patients refractory to pemetrexed.^[5,6]^ However, the effects of immune checkpoint inhibitors on the progression of peritoneal mesothelioma have not been reported. We administered nivolumab to this patient as the second-line therapy to emulate pleural mesothelioma treatment. However, unfortunately, the growth rate of the intraperitoneal mass was markedly accelerated following initial nivolumab treatment.

Hyperprogressive disease, characterized by paradoxically accelerated tumor progression, has recently been proposed as a new pattern of progression after immune checkpoint inhibitor treatment, although its definition has not yet been clearly established.^[13]^ While the mechanisms underlying the development of hyperprogressive disease after immune checkpoint inhibitor treatment have not been fully elucidated, some immunological explanations have been proposed, including the expansion of programmed cell death-1^+^ regulatory T cells.^[14]^ No biological markers capable of predicting hyperprogressive disease have been identified. Hyperprogressive disease during immune checkpoint inhibitor therapy is known to be a relatively common phenomenon, with reported incidence rates of 8% to 14% in non-small cell lung cancer,^[15,16]^ but a case of hyperprogressive disease in mesothelioma has not been reported.

We herein report a man with peritoneal mesothelioma, a rare malignant tumor. He responded well to cisplatin plus pemetrexed treatment but experienced hyperprogressive disease after nivolumab administration. To our knowledge, this is the first report to show hyperprogressive disease in a case of peritoneal mesothelioma after nivolumab treatment. While immune checkpoint inhibitors may be promising therapeutic strategies for treating malignant peritoneal mesothelioma, careful monitoring such as careful follow-up of image findings must be practiced with their application.

## Author contributions

Hiroaki Ikushima, Toshio Sakatani, and Kazuhiro Usui designed the therapeutic methods, performed the treatment, analyzed the data, and wrote the manuscript.

Sayaka Ohara and Hideyuki Takeshima performed the treatment.

Hajime Horiuchi and Teppei Morikawa analyzed the data.
